# The OCT RNFL Probability Map and Artifacts Resembling Glaucomatous Damage

**DOI:** 10.1167/tvst.11.3.18

**Published:** 2022-03-15

**Authors:** Sol La Bruna, Anvit Rai, Grace Mao, Jennifer Kerr, Heer Amin, Zane Z. Zemborain, Ari Leshno, Emmanouil Tsamis, Carlos Gustavo De Moraes, Donald C. Hood

**Affiliations:** 1 Department of Psychology, Columbia University, New York, NY, USA; 2 Albert Einstein College of Medicine, New York, NY, USA; 3 Vagelos College of Physicians and Surgeons, New York, NY, USA; 4 Department of Biomedical Engineering, Duke University, Durham, NC, USA; 5 Bernard and Shirlee Brown Glaucoma Research Laboratory, Department of Ophthalmology, Columbia University Irving Medical Center, New York, NY, USA; 6 Sackler Faculty of Medicine, Tel Aviv University, Tel Aviv, Israel

**Keywords:** glaucoma, optical coherence tomography, artifact

## Abstract

**Purpose:**

The purpose of this study was to improve the diagnostic ability of the optical coherence tomography (OCT) retinal nerve fiber layer (RNFL) probability (p-) map by understanding the frequency and pattern of artifacts seen on the p-maps of healthy control (HC) eyes resembling glaucomatous damage.

**Methods:**

RNFL p-maps were generated from wide-field OCT cube scans of 2 groups of HC eyes, 200 from a commercial normative group (HC-norm) and 54 from a prospective study group, as well as from 62 patient eyes, which included 32 with early glaucoma (EG). These 32 EG eyes had 24-2 mean deviation (MD) better than −6 dB and perimetric glaucoma as defined by 24-2 and 10-2 criteria. For the HC groups, “glaucoma-like” arcuates were defined as any red region near the temporal half of the disc.

**Results:**

Seven percent of the 200 HC-norm and 11% of the 54 HC RNFL p-maps satisfied the definition of “glaucoma-like,” as did all the patients’ p-maps. The HC p-maps showed two general patterns of abnormal regions, “arcuate” and “temporal quadrant,” and these patterns resembled those seen on some of the RNFL p-maps of the EG eyes. A “vertical midline” rule, which required the abnormal region to cross the vertical midline through the fovea, had a specificity of >99%, and a sensitivity of 75% for EG and 93% for moderate to advanced eyes.

**Conclusions:**

Glaucoma-like artifacts on RNFL p-maps are relatively common and can masquerade as arcuate and/or widespread/temporal damage.

**Translational Relevance:**

A vertical midline rule had excellent specificity. However, other OCT information is necessary to obtain high sensitivity, especially in eyes with early glaucoma.

## Introduction

Optical coherence tomography (OCT) has become an invaluable tool in the diagnosis of glaucoma.^1^^–^^14^ As retinal nerve fiber layer (RNFL) thinning is a key component of glaucoma, imaging techniques like OCT have allowed for the visualization and quantification of RNFL changes. Commercially available summary statistics of RNFL thickness are frequently used by clinicians to inform diagnostic decisions.^7^^,^^15^^–^^18^ However, there are shortcomings to relying on these summary statistics.^10^^,^^19^^–^^26^

OCT imaging has allowed for the creation of three-dimensional RNFL thickness maps. From these thickness maps, commercial software generates RNFL probability (p-) maps by comparing a patient's local RNFL thickness values to those of an age-similar healthy control (HC) group. The p-maps have shown clinical utility in the diagnosis of glaucoma.^6^^,^^13^^,^^27^^–^^36^ Recently, these maps have been incorporated into a scheme to define and teach how to use OCT to detect damage based solely upon an OCT report.^37^^,^^38^ Additionally, recent work in artificial intelligence (AI) suggests that the RNFL p-map is more useful than RNFL thickness maps, or than ganglion cell layer thickness or p-maps for differentiating healthy eyes from those with glaucoma.^39^^–^^41^

Although RNFL p-maps appear to be clinically useful, they are not immune to artifacts that can resemble damage due to glaucoma. For instance, it has been documented that confusion can arise due to arcuate artifacts on RNFL p-maps; these artifacts are examples of so-called “red disease,”^42^ and are due to normal variations in the location of the major temporal blood vessels,^10^^,^^23^^,^^43^ and the associated major RNFL bundles.^20^^,^^44^ Unlike artifacts due to scanning or alignment errors, these arcuate artifacts are often confused with “real” arcuate defects caused by glaucomatous damage. Whereas the existence of these glaucoma-like artifacts is generally accepted,^10^^,^^42^^,^^43^ less is known about their frequency. In addition, it is possible that there are other causes for patterns of seemingly abnormal regions on the RNFL p-maps that can also be confused with glaucomatous damage.

For the RNFL p-map to have high specificity, it is important to understand the nature and frequency of artifacts that can be confused with changes due to glaucoma. In this study, we seek to improve the use of the RNFL p-map by comparing artifacts seen on the RNFL p-maps of HC eyes with patterns of glaucomatous damage seen on RNFL p-maps of eyes with glaucoma of ranging severity. In particular, we address three questions: (1) what is the frequency of artifacts on HC RNFL p-maps that might be confused with abnormal patterns seen on the p-maps of patients? (2) what do these patterns look like? Finally, (3) we ask if a simple rule can help to distinguish between the patterns seen on HC artifacts and glaucomatous damage.

## Methods

Study procedures followed the tenets of the Declaration of Helsinki and Health Insurance Portability and Accountability Act and were approved by the Institutional Review Board of Columbia University. Written informed consent was obtained from all participants.

### Participants

The OCT data came from two sources. First, there were 145 eligible eyes from an observational, prospective, case-control study, the Macular Damage in Early Glaucoma and Progression Study (MAPS; PI: C Gustavo De Moraes; ClinicalTrials.gov Identifier: NCT02547740). All 145 eyes had at least 2 baseline OCT scans and 24-2 and 10-2 visual field (VF) within 13 days and had best-corrected visual acuity of 20/40 or better and refractive error between −6.00 and +6.00 diopters (D; spherical equivalent), the typical inclusion criterion for commercial normative groups. These 145 eyes included 54 HC eyes, 61 eyes with early glaucoma or suspected glaucoma (S/EG) with 24-2 VF mean deviation (MD) better than −6 dB from MAPS, as well as contralateral eyes of MAPS that included 12 eyes with moderate glaucoma (MG, 24-2 VF MD >−6 dB and <−12 dB), and 18 eyes with advanced glaucoma (AG; 24-2 VF MD >−12 dB). Four eyes were excluded from an initial group of 149 eyes as they had epiretinal membranes that affected OCT imaging.

All HCs had intraocular pressure (IOP) within statistically normal limits and normal fundus examination. These eyes had multiple 24-2 and 10-2 VFs, as well as OCT tests. Although the inclusion of these eyes was strictly based upon IOP and fundus examination, the OCT and VF tests were confirmatory. All patient eyes had a glaucoma, or glaucoma suspect, diagnosis based upon the referring glaucoma specialist's interpretation of functional (24-2 and 10-2 VFs) and structural (fundus photographs and OCT) information, as well as IOP and clinical history. All eyes had 24-2 and 10-2 VF tests with SITA-Standard protocol (Carl Zeiss Meditec, Inc., Dublin, CA) and OCT scans as described below.

To reduce the number of suspect eyes in the analysis and to form a group of eyes likely to have glaucoma based upon strictly functional information, we applied a modified Ocular Hypertension Treatment Study (OHTS) criteria to the 61 S/EG eyes. An eye was classified as EG_VF_ if three consecutive VFs were reliable VFs and met the criteria below. A reliable field was defined as one with false-positive (FP) errors and false-negative (FN) errors less than 15%, and with fixation losses less than 33%. Note that 33% is used as the limit for fixation losses for OHTS, in contrast to the HFA's default value of 20%. According to the OHTS protocol, an abnormal 24-2 VF is defined as having a glaucoma hemifield test (GHT) outside of normal limits and/or a pattern standard deviation (PSD) with *P* < 0.05 with the abnormality in the same hemifield across the 3 tests.^45^^,^^46^ The OHTS protocol does not include the 10-2 VF. However, several recent publications have shown that eyes classified as normal by the OHTS criteria can have clear macular damage on the 10-2 VF.^47^^–^^50^ Therefore, we applied a similar classification scheme for the 10-2, in which an abnormal 10-2 VF is defined as having an MD with *P* ≤ 0.05 and/or a PSD with *P* ≤ 0.05 with the abnormality in the same hemifield across the three tests. Of the 61 eyes, 32 had 3 consecutive abnormal 24-2 VFs or 3 consecutive abnormal 10-2 VFs (EG_VF_).

**Figure 1.**
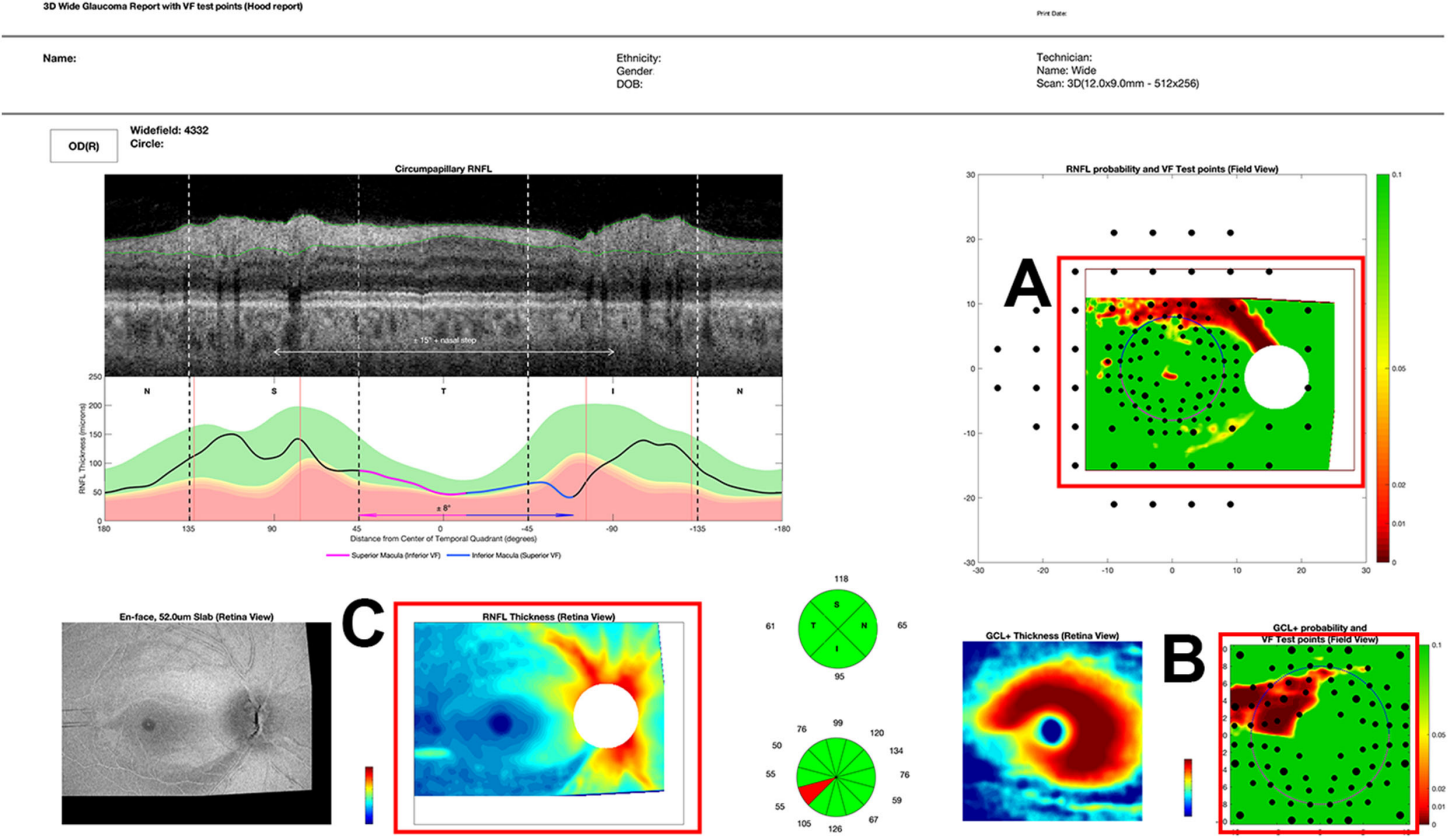
A research version of the commercially available Hood Report based upon a wide-field swept-source OCT volume scan. (**A**) The retinal nerve fiber layer probability map. (**B**) The ganglion cell plus inner plexiform layer probability map. Note that the probability maps are shown in field view so that the top of each corresponds to the upper visual field/inferior retina. (**C**) The RNFL thickness map, in retina view. See references for more details.^10^^,^^33^

An additional 200 healthy eyes (HC-norm) were included from a commercial normative reference group (data provided by Topcon Inc., Tokyo, Japan). These were the first 200, after excluding 2 eyes that had OCT reports consistent with optic neuropathy. In addition to arcuate defects seen on the p-maps, the en-face map, as in the lower left panel of Figure 1, showed a clear arcuate defect. Given the prevalence of glaucoma in the general populations,^51^^,^^52^ and the fact that the 24-2 VF can miss this type of glaucomatous damage,^47^^,^^48^ it is not surprising to find that 2 eyes, 1% among the 200 eyes, might have glaucoma. Because our purpose here was to understand “true” arcuates, these two eyes were excluded.

### Optical Coherence Tomography

Wide-field (9 × 12 mm) swept-source OCT volume scans (Topcon, Inc.) were obtained for each eye, consisting of 256 B-scans, each with 512 A-scans. For the 145 MAPS eyes, in all but 1 case, the scan came from the first baseline visit. In that one case, the first baseline image suffered from poor focusing, so the second visit was used instead. In most cases, there was more than one scan available on the first baseline visit, so we used the same scan as the Sun et al. study.^53^ In that study, every report and scan underwent quality assessment by two OCT experts/readers to identify the scan with the best quality from each study visit. Scans with incorrect centering, significant eye motion, or blink artifacts that would result in loss of measurements from the disc or macula were excluded. It is important to note that the exclusion criteria did not either directly or indirectly include artifacts that could be confused with glaucoma-like damage, which are the focus of the present study.

For the 200 HC-norm eyes, there was only one scan available per eye.

From the widefield scans, thickness values of the RNFL were extracted. A 6 × 6 mm region of the wide-field scan, which was centered on the fovea, was used to obtain the thickness values of the retinal ganglion cell plus inner plexiform layer (GCL+) of the macular region. Using the reference database from the OCT device manufacturers, we generated age-corrected p-maps, used in an established OCT wide-field report that is commercially available outside of the United States. Figure 1 provides an example of the OCT wide-field report. This study focused predominantly on the two framed p-maps: the RNFL p-map (A) and the GCL+ p-map (B) in Figure 1. Both p-maps are in field view so that the upper region corresponds to the superior VF/the inferior retina. The symbols seen on the p-maps in all figures indicate the location of the 24-2 (larger symbols) and 10-2 (smaller symbols) VF test points.

### Identifying Glaucoma-Like Artifacts on HC RNFL p-Maps

**Figure 2.**
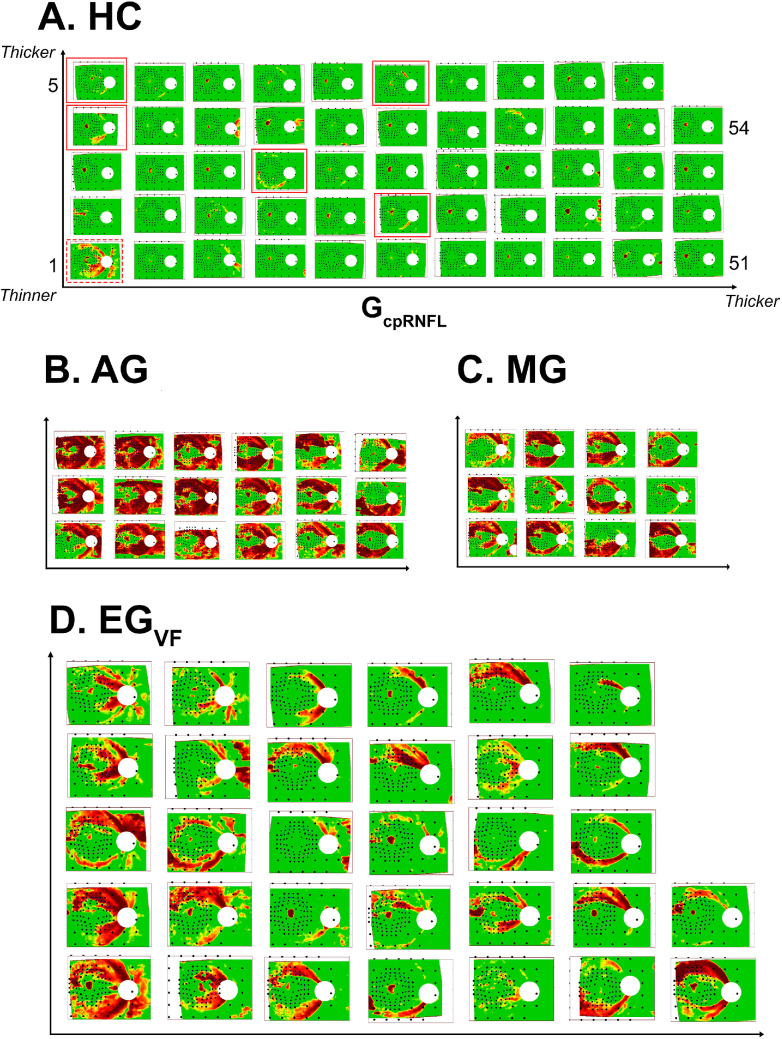
The retinal nerve fiber layer (RNFL) probability (p-) maps ordered by global circumpapillary RNFL thickness (G_cpRNFL_) for each of the groups. For each panel, the thinnest G_cpRNFL_ is in the lower left and the thickest is in the upper right. (**A**) The RNFL p-maps ordered by G_cpRNFL_ for the 54 healthy controls (HCs). (**B**) Same as panel **A**, but for the 18 advanced glaucoma (AG) eyes. (**C**) Same as panel **A**, but for the 12 moderate glaucoma (MG) eyes. (**D**) Same as panel **A**, but for the 32 early glaucoma eyes (EG_VF_) that meet the modified Ocular Hypertension Treatment Study (OHTS) criteria.

To identify glaucoma-like abnormal patterns on the RNFL p-maps, we assumed that a red region near the temporal half of the disc is a necessary condition to classify an RNFL p-map as “abnormal.” This necessary condition for glaucoma was based upon the observation documented below that all the patients with glaucoma met this condition. Using this definition, 3 authors (S.L.B., A.R., and D.C.H.) separately scrutinized the 54 HC p-maps and the 200 HC-norm p-maps. In instances of disagreement, there was adjudication, and a consensus was reached. In the case of the 54 HC from MAPS, all the p-maps are presented below so the readers can make their own judgment (Fig. 2A).

### Metric Analyses

To compare the HC eyes with glaucoma-like artifacts to the most commonly used summary statistic of RNFL thickness, for each eye, we calculated the global (average) circumpapillary (cpRNFL) thickness (G_cpRNFL_) using the wide-field OCT scan and derived B-scan image for the 3.4-mm diameter circle, as previously described.^50^

## Results

Based upon the inclusion criteria discussed above, the analysis of RNFL p-maps included 54 HC, 32 EG_VF_, 12 MG, 18 AG, and 200 HC-norm eyes. Figure 2 shows the RNFL p-maps for the first 4 of these groups.

### Identifying Artifacts in HCs that may be Confused With Glaucomatous Damage

The RNFL p-maps in Figure 2 are ordered based upon the G_cpRNFL_. For example, for the 54 HC eyes (see Fig. 2A), the thinnest G_cpRNFL_ (#1) is in the lower left and the thickest (#54) is in the upper right. We chose G_cpRNFL_ as it is a common OCT measure of severity of damage.^16^^,^^35^^,^^54^^–^^62^

**Figure 3.**
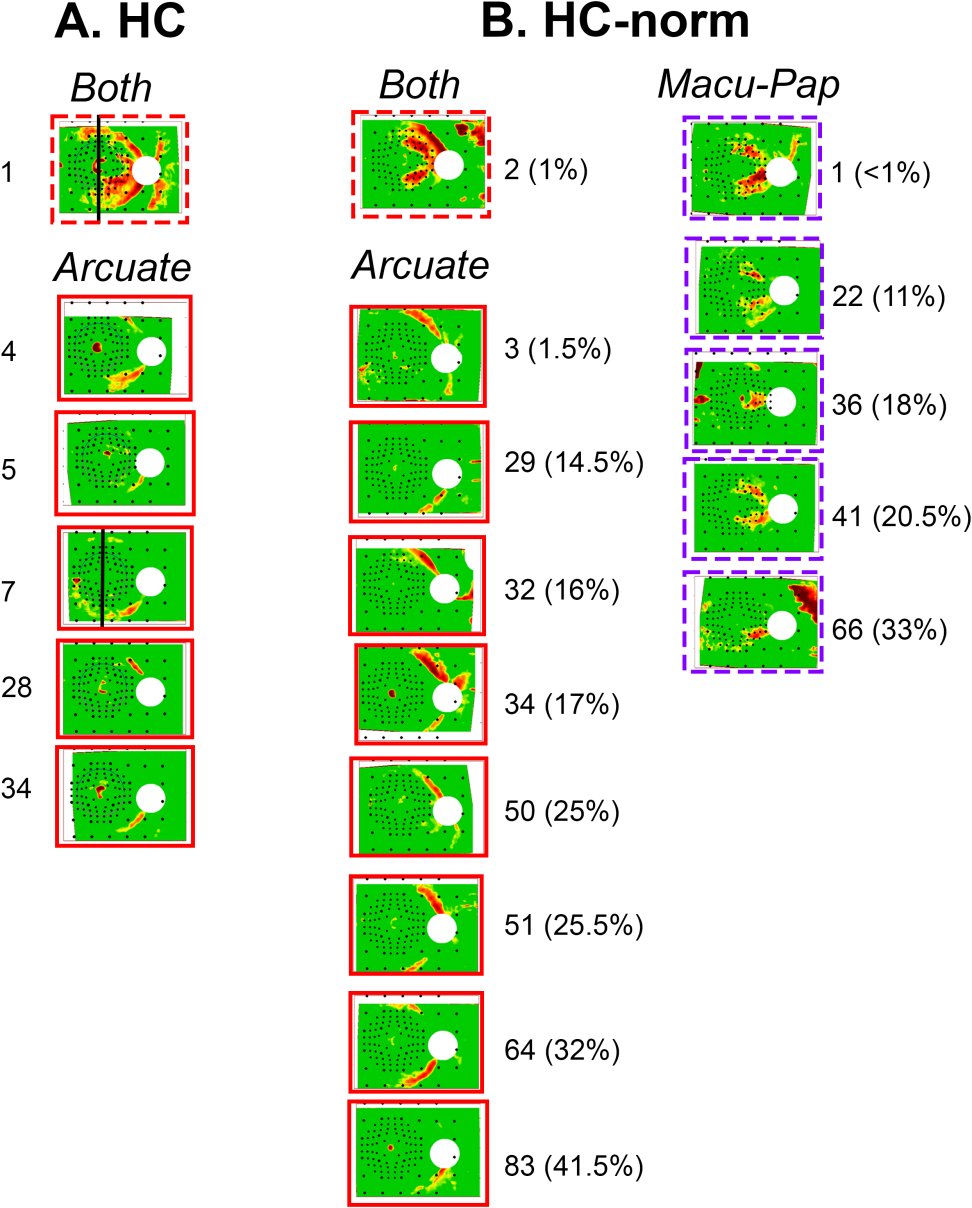
The RNFL p-maps of HC eyes that could be mistaken for glaucoma. (**A**) The 6 RNFL p-maps of the 54 HC with an “arcuate” artifact (*solid red border*) or with both a “temporal quadrant (Q)” and “arcuate” artifact (*dashed red border*). The numbers next to the RNFL p-maps describe the position in the distribution as the *n*th thinnest (i.e. for the RNFL p-map in the top left, it is the thinnest [first], whereas the one below is the fourth thinnest). The *black line* on two of the RNFL p-maps denote the vertical midline through the fovea. (**B**) The 14 RNFL p-maps of the 200 healthy controls from a commercial normative database (HC-norm) that have an “arcuate” artifact (*red border*), a “temporal (Q)” artifact (*dashed purple border*), or both (*dashed red border*). The numbers next to the RNFL p-maps describe the position in the distribution as the *nt*h thinnest, as well as its percentile position in the distribution. For example, the first p-map in the top left column is the second thinnest and is in the first percentile.

Notice that all the 62 AG/MG/EG_vf_ eyes in panels B to D of Figure 2 have a red region near the temporal side of the disc, consistent with our working hypothesis, which assumed that a red region near the temporal half of the disc is a necessary condition to classify an RNFL p-map as abnormal. Although this appears to be a necessary condition, it is not sufficient as 6 (11%) of the RNFL p-maps of the 54 HCs met this condition. These 6 p-maps have a red border in Figure 2A and enlarged versions of these 6 p-maps are shown in Figure 3A. These are the HC RNFL p-maps that the clinician might confuse with those from eyes with glaucomatous damage.

To enlarge our sample of HCs with artifacts, we examined 200 RNFL p-maps from a commercial database mentioned in the Methods. Of these 200, 14 (7%) had artifacts defined as a red region on the temporal half of the disc. The RNFL p-maps for these 14 are shown in Figure 3B.

### Classification of RNFL p-Map Artifacts in HC

The artifacts identified in the HC RNFL p-maps appeared to fall into three groups (as indicated in Figs. 3A,B): (1) “arcuate”: an arcuate-shaped artifact that could be mistaken for arcuate damage commonly seen in glaucoma; (2) “temporal quadrant (Q)”: artifacts that include the temporal Q of the disc and the maculo-papillary region of the p-map; they can be mistaken for maculo-papillary and/or diffuse glaucomatous damage; and (3) “both”: artifacts that appeared to be a combination of the two. The Table shows the number of cases for each type of artifact for the HC and HC-norm groups.

**Table.** Total Number of Glaucoma-Like Artifacts in HCs

Group	Arcuate	Temporal Q	Both
HC-norm (*n* = 200)	8	5	1
HC (*n* = 54)	5	0	1
Total (*n* = 254)	13 (5%)	5 (2%)	2 (<1%)

HC, healthy control; HC-norm, healthy control commercial normative group.

### A “Vertical Midline” Rule for Identifying HC Artifacts

To minimize FP results and obtain high sensitivity, we assumed as a working hypothesis that the abnormal region on the RNFL p-map must cross the midline (the black vertical lines through the center of the fovea in Figs. 3A,B). The anatomic basis for this “vertical midline rule” has to do with the observation that arcuate artifacts seen on RNFL p-maps of HCs are associated with displacement of the major temporal arcuate bundles of the RNFL.^10^^,^^23^ Notice in Figure 3A and B that the abnormal red/yellow region crosses the midline (black vertical line in Fig. 3A) in only 2 of the 15 RNFL p-maps with “arcuate” artifacts. Although it is likely that the “temporal Q” artifacts are due to more than one deviation from normal anatomy, they too do not cross the midline in the RNFL p-map of HCs. In fact, none of the 5 “temporal Q” artifacts in Figure 3B cross the midline.

### The Sensitivity of the Vertical Midline Rule

**Figure 4.**
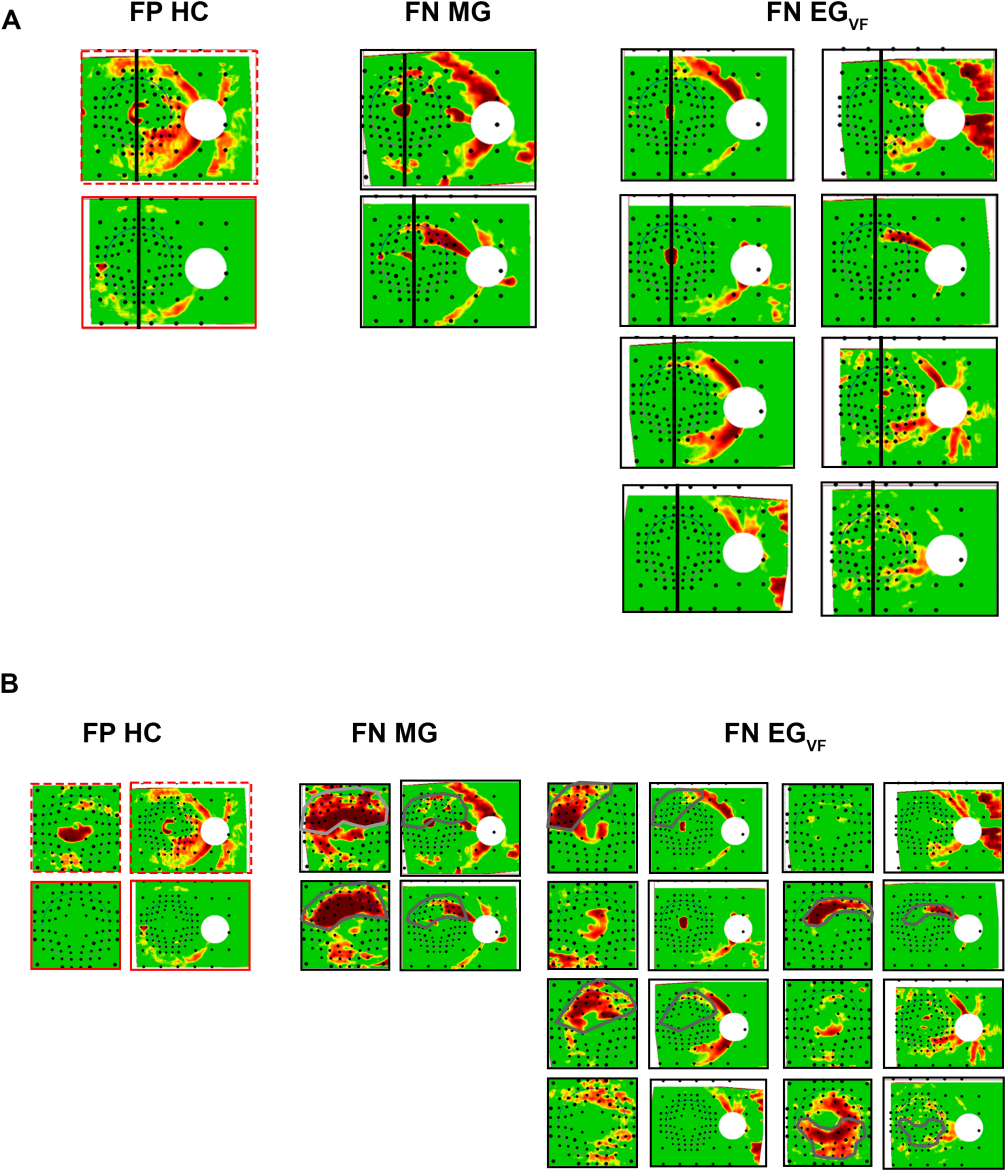
(**A**) The RNFL p-maps of FP results and FN results based on the midline rule. RNFL p-maps that have an “arcuate” artifact (red border), or both an “arcuate” and “temporal Q” artifact, are outlined (*dashed red border*), whereas patient eyes are outlined with *black borders*. The *black line* on each RNFL p-map demarcates the vertical midline through the fovea. (**B**) The same, but the RNFL p-maps are accompanied by their corresponding ganglion cell plus inner plexiform layer (GCL+) p-map. Those patient eyes with topographically correspondent damage on the RNFL and GCL+ p-map have arcuate lines demonstrating the regions of damage.

All of the AG, and all but two of the MG, RNFL p-maps showed abnormal arcuate regions that cross the midline, for a sensitivity of 93% for the combined MG/AG eyes. On the other hand, the sensitivity for the EG_VF_ group was only 75%, as 8 of the 32 failed the midline test. Thus, based upon the vertical midline rule, there were 2 FP results (99.2% of 254, HC plus HC-norm) and 10 FN results (84% of the 62 AG/MG/EG_vf_). Figure 4A shows the RNFL p-maps for these FP and FN results. Notice that these p-maps overlap in appearance.

### Relation of RNFL p-Map Artifacts to cpRNFL Thickness

**Figure 5.**
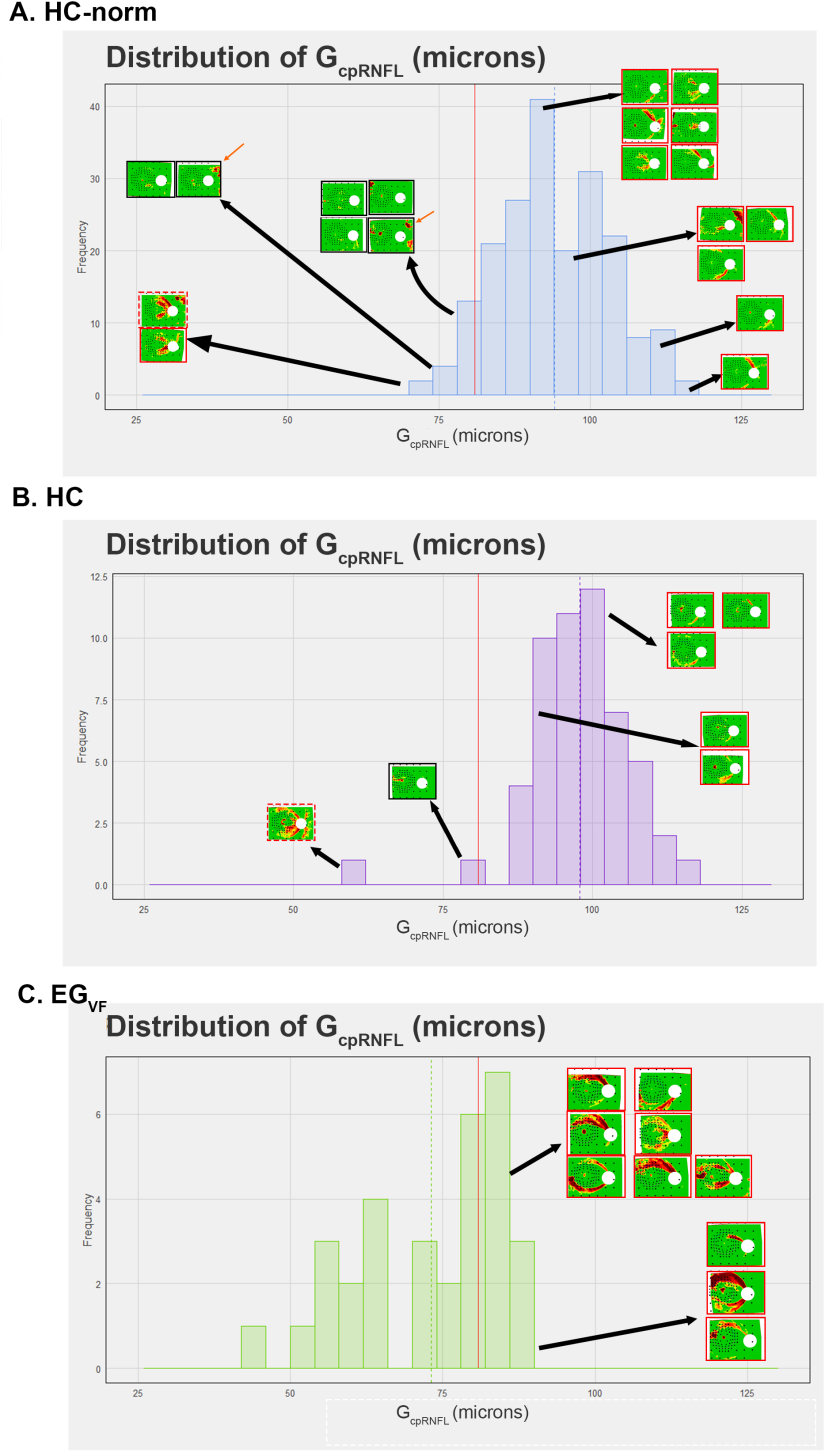
Histogram distributions of G_cpRNFL_ thickness for the (**A**) HC-norm, (**B**) HC, and (**C**) EG_VF_. The *red line* provides the bottom fifth percentile threshold calculated from the HC-norm. The *dashed line* on each panel provides the average G_cpRNFL_ for each group. The position of the individual p-maps along the distributions are depicted by *black arrows*. The *red*, *solid borders* and *dashed red borders* surrounding p-maps provide examples of eyes that, in **A** and **B**, could be mistaken as glaucomatous (the *solid red border* indicates “arcuate” artifacts and the *dashed red borders* indicate both “temporal Q” and “arcuate” artifacts), and in (**C**), that have glaucoma. The RNFL p-maps with *black borders* in **A** and **B** are eyes that are unambiguously normal. The *orange arrows* in **A** point to instances in which there are scanning artifacts. In **A** and **B**, eyes on the left of the *red line* are false positive results (FPs), whereas eyes on the right of the *red line* in **C** are false negative results (FNs).

As G_cpRNFL_ thickness is a commonly used single OCT measure of RNFL damage, we asked if it was a good predictor of the artifacts seen in RNFL p-maps. Figure 5 shows a histogram of the distribution of G_cpRNFL_ thicknesses for the HC (A), HC-norm (B), and EG_VF_ (C) groups. The vertical dotted line within each panel indicates the average G_cpRNFL_ thickness for that group, and the red vertical line represents the 5% lower percentile calculated from the HC-norm. For the HC groups in panels A and B of Figure 5, eyes falling to the left of this vertical red line are FP results.

There are two aspects of note in panels A and B of Figure 5. First, the RNFL p-maps of some of the G_cpRNFL_ FP results are unambiguously normal. For example, the RNFL p-maps within the black boxes have little or no red region, or a red region that is clearly not due to glaucoma (e.g. small red regions near fixation or scanning artifacts, orange arrows). Second, some of the HC RNFL p-maps with arcuate artifacts, which the clinician might mistake for glaucomatous damage, have G_cpRNFL_ thicknesses well above the 5th percentile of the HC-norm, as indicated by the insets with red borders in Figure 5A and B and the percentiles in parentheses in Figure 3B.

## Discussion

As clinicians increasingly rely on OCT RNFL p-maps, it is important to understand the artifacts in RNFL p-maps of HCs. Whereas previous work on artifacts have generally focused on scanning artifacts due to eye movements, segmentation errors, and centering,^22^^,^^24^^,^^25^^,^^63^^–^^69^ we focused on glaucoma-like artifacts. As detailed below, we addressed three questions. First, what was the frequency of artifacts on HC RNFL p-maps that could be confused with abnormal patterns seen on the p-maps of patients? Second, what do the p-maps of HCs that could be mistaken for glaucoma look like? Finally, we asked if a simple rule, the vertical midline rule, could help to distinguish between the HC artifacts and glaucomatous damage.

### The Frequency and Patterns of Glaucoma-Like Artifacts

About 11% of the HC p-maps had artifacts resembling the general patterns seen in patients with glaucoma. Further, we found that these glaucoma-like artifacts take on two general forms: (1) “arcuate”: an arcuate-shaped artifact that could be mistaken for arcuate damage commonly seen in glaucoma; and (2) “temporal Q”: artifacts associated with the temporal quadrant of the disc and that could be mistaken for maculo-papillary damage and/or diffuse damage. A third, and less frequent, group includes eyes with both “arcuate” and “temporal Q” artifacts.

“Arcuate” artifacts were the most common type of artifact, and previous work suggests that these artifacts may be caused by anatomic variations in the location of blood vessels and the associated RNF bundles.^10^^,^^20^^,^^23^^,^^43^^,^^44^ “Temporal Q” artifacts were less common and proved more difficult to distinguish from HCs, as discussed next.

### The Vertical Midline Rule

We hypothesized that “arcuate” artifacts should not cross the midline, defined as a vertical line through the fovea (see Fig. 2A, Fig. 4A). This vertical midline rule is based upon the observation that arcuate artifacts seen on RNFL p-maps of HCs are associated with displacement of the major temporal arcuate bundles of RNFL, whereas true glaucomatous damage follows arcuates that cross the midline outside the maculo-papillary bundle. In any case, the rule, as expected, had excellent specificity. Only 2 of the 254 HC eyes crossed the midline. That is, the specificity was about 99%.

### Beyond the Vertical Midline Rule

On the other hand, the vertical midline rule missed glaucomatous damage, especially in EG_VF_ eyes. Out of the 62 AG/MG/EG_vf_ eyes, there were 10 FN results (84% sensitivity) with 8 of these 10 being EG_vf_ eyes for a sensitivity of 75% for EG_vf_ eyes. It is possible that other aspects of the patterns seen on RNFL p-maps can be used to distinguish between HCs and EG_vf_ eyes. For example, a visual examination of the EG_vf_ p-map patterns in Figure 2D, which do not cross the vertical midline, suggests that they tend to have deeper, wider, and more extensive abnormal regions than the HC artifacts in Figure 3. However, there is an overlap in appearance that will make it difficult for a clinician to reliably use this information. It is possible, however, that an AI program based upon RNFL p-maps, such as that developed by Thakoor and colleagues,^40^^,^^70^^,^^71^ may be able to use this information.

However, it is important to note that the vertical midline rule *only* uses the OCT RNFL p-map. Other information on the OCT report in Figure 1 can help to distinguish between eyes with early glaucoma and HCs.^37^^,^^38^ As an illustration, in Figure 4B we show the GCL+ p-maps for the FP results and FN results in Figure 4A. For at least 6 of the 10 FN results, GCL+ p-maps clearly confirm that the arcuate seen on the RNFL p-map is consistent with glaucoma. Note in these six eyes, the combined abnormal region on the GCL+ and RNFL p-maps does cross the midline (Fig. 4B). See figure caption for details.

### Limitations

Our study has the following limitations. First, a similar analysis should be made for other OCT models and manufacturers. It is unlikely that the manufacturer or make of the OCT instrument per se will be a significant factor, as the major source of these artifacts is anatomic variation among the HC eyes. On the other hand, the frequency of the glaucoma-like defects will certainly be impacted by the composition of the control group, as well as the software used to produce the p-maps from thickness maps; both control group composition and methodology for producing p-maps vary across manufacturers.

Another limitation is the sample size of the glaucomatous eyes. A replication with a larger sample size of glaucomatous eyes of varying severities should be performed. Additionally, this study did not include eyes with high myopia, although it should be possible to analyze the p-maps in many eyes with high myopia.^72^

It might be argued that the use of the modified OHTS criteria to select EG eyes is a limitation. In fact, this may have resulted in the elimination of some EG eyes with even more subtle damage than seen in the EG_vf_, although it is not clear how this would impact the conclusions here. In any case, we did not want to confuse the analysis with EG eyes that were in fact healthy. Further, we could argue that the use of functional inclusion criteria to examine structural data is a strength of the design.

Finally, our analysis did not take into consideration that these artifacts may be present in eyes with glaucoma as well. If, as we suspect, anatomic variation is the source of these artifacts, then they should be present in approximately the same percentage in eyes with EG. For example, we should expect about 3 (10%) of the 32 EG_vf_ eyes to be influenced. Although given the patterns we see in the HC p-maps in Figure 2, in most cases. the impact should be minor.

## Conclusions

Glaucoma-like artifacts on RNFL probability maps are relatively common and can masquerade as arcuate and/or widespread and/or temporal damage. These artifacts are characterized by a failure for the abnormal region to cross the vertical midline. Although the vertical midline rule had excellent specificity, other OCT information is necessary to avoid FN results and to obtain high sensitivity, especially in eyes with early glaucoma.
